# 1285. Risk Factors for Infection Following Revision Hip and Knee Arthroplasty

**DOI:** 10.1093/ofid/ofad500.1124

**Published:** 2023-11-27

**Authors:** Joseph DeBiase, Ryan Carroll, Nora Colburn, Shandra R Day

**Affiliations:** Ohio State University Wexner Medical Center, Columbus, Ohio; The Ohio State University, Columbus, Ohio; The Ohio State University Wexner Medical Center, Columbus, Ohio; Ohio State University Wexner Medical Center, Columbus, Ohio

## Abstract

**Background:**

Patients undergoing revision total hip (THA) and knee (TKA) arthroplasties are at increased risk for surgical site infections (SSI) with prior infection representing a common indication for revision. Our aim was to evaluate patient characteristics and risk factors in patients with THA and TKA infection following revision arthroplasty.

**Methods:**

A single center retrospective review of patients with THA or TKA SSIs from 01/01/2017 to 11/01/2022 was performed utilizing National Healthcare Safety Network (NHSN) definitions and surgical revision codes. Index surgery was defined as the THA or TKA to which the SSI was attributed per NHSN guidelines. Patients meeting NHSN PATOS (present at time of surgery) definition were excluded. Pertinent demographics data, surgical histories and microbiology were collected and the determination of new vs. persistent infection was made based on microbiologic data.

**Results:**

Forty-seven cases were included with median age of 62, 45% male and 51% current/former smokers. BMI was ≥35 in 36% of patients, with 33% having diabetes mellitus and 13% were immunocompromised. Twenty-seven (57%) patients had a prior infection at the involved joint with 18/27 (67%) occurring within 1 year of the index surgery. Table 1 includes index surgery details and infection data. Median time to infection was 25 days. Leading pathogens were *S. aureus* 16 (34%), *Enterococcus* species 11 (23%), enterobacterales 10 (21%), and polymicrobial infections 10 (21%).

**Figure:**
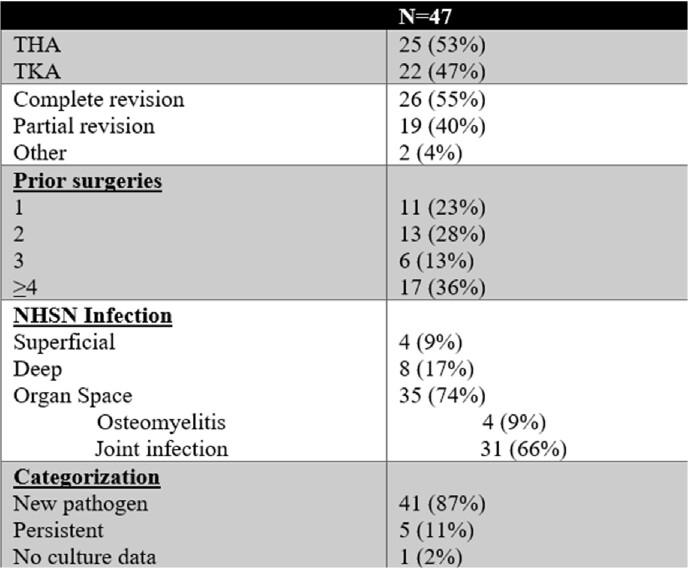

**Conclusion:**

Over half of patients that developed revision THA/TKA SSI had a prior infection but less than 15% had a persistent infection. Most patients had multiple prior surgeries which may contribute to longer operative times and delayed wound healing. *S. aureus* was the most common pathogen identified but many cases involved less typical SSI pathogens. These results highlight the importance of focusing on patient specific risk factors, surgical infection prevention strategies and wound care, rather than additional pathogen specific antibiotics. While more study is needed to delineate SSI risk factors following revision arthroplasty, our study highlights the multitude of potential factors involved in these highly morbid infections.

**Disclosures:**

**All Authors**: No reported disclosures

